# Circulating Cholesterol Levels May Link to the Factors Influencing Parkinson’s Risk

**DOI:** 10.3389/fneur.2017.00501

**Published:** 2017-09-27

**Authors:** Lijun Zhang, Xue Wang, Ming Wang, Nick W. Sterling, Guangwei Du, Mechelle M. Lewis, Tao Yao, Richard B. Mailman, Runze Li, Xuemei Huang

**Affiliations:** ^1^Institute for Personalized Medicine, Pennsylvania State University College of Medicine-Milton S. Hershey Medical Center, Hershey, PA, United States; ^2^Department of Industrial and Manufacturing Engineering, Pennsylvania State University, University Park, PA, United States; ^3^Department of Public Health Sciences, Pennsylvania State University College of Medicine-Milton S. Hershey Medical Center, Hershey, PA, United States; ^4^Department of Neurology, Pennsylvania State University College of Medicine-Milton S. Hershey Medical Center, Hershey, PA, United States; ^5^Department of Pharmacology, Pennsylvania State University College of Medicine-Milton S. Hershey Medical Center, Hershey, PA, United States; ^6^Department of Statistics, Pennsylvania State University, University Park, PA, United States; ^7^Department of Radiology, Pennsylvania State University College of Medicine-Milton S. Hershey Medical Center, Hershey, PA, United States; ^8^Department of Neurosurgery, Pennsylvania State University College of Medicine-Milton S. Hershey Medical Center, Hershey, PA, United States; ^9^Department of Kinesiology, Pennsylvania State University College of Medicine-Milton S. Hershey Medical Center, Hershey, PA, United States

**Keywords:** Parkinson’s disease, cholesterol, genetics, statins, propensity score

## Abstract

**Objectives:**

A growing literature suggests that circulating cholesterol levels have been associated with Parkinson’s disease (PD). In this study, we investigated a possible causal basis for the cholesterol-PD link.

**Methods:**

Fasting plasma cholesterol levels were obtained from 91 PD and 70 age- and gender-matched controls from an NINDS PD Biomarkers Program cohort at the Pennsylvania State University College of Medicine. Based on the literature, genetic polymorphisms in selected cholesterol management genes (APOE, LDLR, LRP1, and LRPAP1) were chosen as confounding variables because they may influence both cholesterol levels and PD risk. First, the marginal structure model was applied, where the associations of total- and LDL-cholesterol levels with genetic polymorphisms, statin usage, and smoking history were estimated using linear regression. Then, potential causal influences of total- and LDL-cholesterol on PD occurrence were investigated using a generalized propensity score approach in the second step.

**Results:**

Both statins (*p* < 0.001) and LRP1 (*p* < 0.03) influenced total- and LDL-cholesterol levels. There also was a trend for APOE to affect total- and LDL-cholesterol (*p* = 0.08 for both), and for LRPAR1 to affect LDL-cholesterol (*p* = 0.05). Conversely, LDLR did not influence plasma cholesterol levels (*p* > 0.19). Based on propensity score methods, lower total- and LDL-cholesterol were significantly linked to PD (*p* < 0.001 and *p* = 0.04, respectively).

**Conclusion:**

The current study suggests that circulating total- and LDL-cholesterol levels potentially may be linked to the factor(s) influencing PD risk. Further studies to validate these results would impact our understanding of the role of cholesterol as a risk factor in PD, and its relationship to recent public health controversies.

## Introduction

Although the exact cause for Parkinson’s disease (PD) is unknown, behavioral and environmental factors have a strong effect on PD risk. A growing literature has provided evidence that circulating cholesterol is related to PD. First, case–control studies have found that higher plasma cholesterol is associated with reduced PD prevalence (1–4). Second, several prospective studies have indicated that low cholesterol predates the diagnosis of PD (5–9). Third, higher baseline cholesterol may be linked to slower PD progression (10), better cognitive and motor performance (11), and delayed age of PD onset (12).

Although these studies show clear and consistent associations, the observed cholesterol–PD relationship may not be causal. For example, PD diagnosis may prime for adoption of a “healthier” lifestyle, thereby leading to lower cholesterol, or indeed, there could be an unknown behavioral (e.g., smoking) or medical (e.g., use of statin) confounder. Alternatively, lower plasma cholesterol simply may reflect metabolic or non-motor changes that are associated with PD. Thus, the current study was designed to investigate a potential causal relationship between the cholesterol–PD link by studying a number of factors that may affect both circulating cholesterol levels and PD risk.

## Materials and Methods

### Cohort Subjects

Parkinson’s disease (*n* = 91) and control (*n* = 70) subjects (hereafter controls) with a Mini-Mental State Exam (MMSE) score ≥26 were selected from an ongoing cohort study that is part of the NINDS PD Biomarkers Program (13) at the Penn State site. PD patients were recruited from the Penn State Hershey Medical Center movement disorders clinic, and Controls free of any major neurological disorders were recruited from the spouse population and local community. PD and Controls were age-, gender-, and education-matched based on overall distribution. PD diagnosis was confirmed using published criteria (14). Subjects with signs of dementia were excluded using the MMSE score cutoff described above. All PD and control subjects also were free of neurological disorders other than PD, and free of major and acute medical issues such as liver, kidney, or thyroid abnormalities, and deficiencies of B12 or folate. All brain images were deemed free of any major structural abnormalities. Blood was collected after an 8–12 h overnight fast at the baseline visit. Although many metabolic factors may influence cholesterol or PD, the role of diabetes in PD is still in debate. In the current study, we collected fasting lipid profiles and statin usage as main factors of interest for this study. We did not assess either diagnosis of diabetes or drugs to treat diabetes, nor did we measure blood glucose or hemoglobin A1c levels. Written informed consent was obtained for all subjects in accordance with the Declaration of Helsinki. The Penn State Hershey Institutional Review Board approved the research study protocol.

### Plasma Cholesterol

Plasma triglyceride, total-, and HDL-cholesterol levels were measured by enzymatic methods as described previously (11). LDL-cholesterol was calculated using the Friedwald equation:

(1) $${\text{LDL}}_{C}={\text{Chol}}_{\text{total}}-\left(\frac{\text{Triglycerides}}{5}+{\text{HDL}}_{C}\right).$$

### Behavioral and Medical Information

Age, gender, statin usage, and history of smoking were obtained as part of a comprehensive demographic survey. Subjects were considered smokers if they had smoked one cigarette per day for 6 months or more at some point in the past (15). Subjects were considered statin users if they were using statins at the time of blood sampling.

### NeuroX Genotyping Data Acquisition and Analysis

Single-nucleotide polymorphism (SNP) genotyping was performed on whole blood DNA samples extracted by the NINDS PDBP using the Illumina NeuroX array. A total 269,476 variants were genotyped, and the Genotyping Analysis Module within Genome Studio version 1.9.4 was used to call participant genotypes. The threshold call rate for sample inclusion was 95%. Concordance between reported sex and determined sex estimated from X chromosome heterogeneity was required for enrollment in the current study. X chromosome heterogeneity calculations were based on common SNPs from the International HapMap Project that had genotypes with missingness <5% and Hardy–Weinberg equilibrium *p* values > 10^–5^. Samples also were excluded if they were six SDs from the population mean rate of heterozygosity based on common polymorphisms (16).

The Klemann et al. (17) study of 13,094 PD and 47,148 controls showed that lipids and lipoproteins are involved functionally in, and affect, dopaminergic neuron-specific signaling cascades, providing insight into how lipids and lipoproteins may play a key role in PD. The APOE and amyloid-β (Aβ) pathways in the brain show APOE, LDLR, LRP1, and LRPAP1 are involved in the cycling of cholesterol and the delivery of lipids (18). Several other studies show that cholesterol recycling may be linked to PD (19–21). APOE and LRPAP1 SNPs are associated significantly with increased PD risk (22), and both APOE and its receptors (LDLR and LRP1) have been shown to be increased in striatal and hippocampal tissue after MPTP lesions (23). Wilhelmus et al. (24) also observed increased APOE and LRP1 expression in PD and Lewy body disease. Together, the literature provided a strong biological rationale to choose these genes for this study.

We extracted the APOE, LDLR, LRP1, and LRPAP1 SNPs from the NINDS NeuroX genotyping data. Within the NeuroX genotyping data, there are 40 allele loci for the LDLR that contain two major mutation SNPs (rs6511720 and rs2228671), 72 allele loci for LRP1 with three mutation SNPs (rs11172113, rs1466535 and rs1800127), and 17 allele loci for LRPAP1 that contain one major mutation SNP (rs11549516). GWAS studies with these SNPs show they have an association with lipids at *p* < 0.0001 (25, 26).

### Statistical Analysis

For comparison of demographic data between PD and controls, Pearson chi-square tests or Fisher’s exact tests were applied for categorical variables (e.g., gender) and two-sample *t*-tests or Wilcoxon rank-sum tests were used for continuous variables (e.g., age), as appropriate in Table 1. The effects of genetic polymorphisms (LDLR, LRP1, LRPAP1, and APOE), age, gender, statin usage, and behavioral factors (smoking) on total- and LDL-cholesterol levels first were analyzed using linear regression analyses for estimating the propensity scores. To determine whether total- and LDL-cholesterol (exposures of interests) had a causal influence on PD occurrence, a marginal structure model with estimated propensity score (27, 28) was used next. Namely, the propensity score of being assigned to the exposure group given a set of observed potential confounders (e.g., genetic polymorphisms, age, gender, and behavioral factors) controls for differences in confounders between the exposure and non-exposure groups (29–31). Recently, Zhu et al. (28) described a boosting algorithm for estimating generalized propensity scores. The generalized propensity score was assumed to depend on a linear combination of the confounders, and the ignorability assumption was imposed such that it was sufficient to condition on the generalized propensity scores in order to adjust for confounding instead of conditioning on the vector of covariates (28).

**Table 1:** Characteristics of the study subjects.

	Control (*N* = 70)	PD (*N* = 91)	*p*-Value
Gender (F:M)	32:38	42:49	0.96^a^
Age (years)	63.5 ± 11.6	65.8 ± 11.2	0.20^b^
Smoke (No:Yes)	49:21	63:28	0.92^a^
Statin Usage (No:Yes)	44:26	62:29	0.48^a^
Total-cholesterol (mg/dL)	195.2 ± 37.1	183.0 ± 33.5	**0.03**^c^
LDL-cholesterol (mg/dL)	118.5 ± 33.2	108.4 ± 26.6	**0.04**^c^
HDL-cholesterol (mg/dL)	52.1 ± 15.7	53.0 ± 15.6	0.72^c^
Triglycerides (mg/dL)	123.4 ± 66.6	108.0 ± 51.0	0.11^c^

**APOE polymorphism**			

ε3ε3:ε3ε2:ε2ε2:ε3ε4:ε4ε4:ε4ε2	56:14:0:0:0:0	78:12:1:0:0:0	
Freq. of APOE genotypes (%)	80:20:0:0:0:0	86:13:1:0:0:0	
No. of APOE ε2 allele	14	12	
No. of APOE ε3 allele	126	168	

**LDLR single-nucleotide polymorphisms (SNPs)**			

rs6511720 (GG TG TT) (T%)	57:12:1 (10%)	66:22:3 (15.4%)	0.45^d^
rs2228671 (CC TC TT) (T%)	60:10:0 (7.1%)	67:21:3 (14.8%)	0.10^d^

**LRP1 SNPs**			

rs11172113 (CC CT TT) (T%)	19:23:28 (56.4%)	17:42:32 (58.2%)	0.19^d^
rs1466535 (CC TC TT) (C%)	32:26:12 (64.3%)	38:43:10 (65.4%)	0.35^d^
rs1800127 (CC TC TT) (T%)	66:3:1 (3.6%)	85:6:0 (3.3%)	0.49^d^

**LRPAP1 SNPs**			

rs11549516 (CC TC TT) (T%)	65:5:0 (3.6%)	89:2:0 (1.1%)	0.24^d^

*^a^Chi-square tests*.

*^b^Two-sample t-tests*.

*^c^Wilcoxon rank-sum tests*.

*^d^Fisher’s exact tests*.

If we let *Y* denote PD status (Yes = 1; No = 0), *M* be the continuous measurement (such as LDL- or total-cholesterol), and *X* be a *p*-dimensional vector of covariates (including genetic markers and environmental factors), then the observed data including *n* random samples can be represented as (*Y_i_, M_i_, X_i_*), *i* = 1, 2, …, *n*. For any value of the *M* from a continuous domain noted by *m*, the corresponding outcome is defined as *Y_i_*(*m*). Given the ignorability assumption described by Zhu et al. (28), the following marginal structural model (MSM) for the binary outcome is

(2) $$\text{logit}(E(Y_{i}(m)))=\alpha_{0}+\alpha_{1}m$$

where the parameters with standard errors (SE) can be estimated by inverse probability weights (IPW). Thus, the estimates of odds ratios with 95% confidence intervals (CIs) can be calculated without conditioning on any covariates. Of note, the weights can be obtained by the generalized propensity score that is the conditional density of observing m given the covariates denoted by *r*(*m, X*) = *f_m_*_|_*_X_*(*m, X*). Here, we consider a multiple regression model for the generalized propensity score, which is traditional and written by

(3) $$M=X^{T}\beta +\varepsilon ,\;\varepsilon ∼N(0,\sigma^{2})$$

where the considered covariates include age, gender, statin usage, smoking, APOE, LDLR, LRP1, and LRPAP1 gene SNPs. Following the procedures of Ji et al. (32), the weight for the *i*th subject is calculated by $w_{i}=\frac{\hat{r}(m_{i})}{\hat{r}(m_{i},X_{i})}$, where $\hat{r}$ is the estimation of the propensity score. The analyses were conducted using R software (version 3.3.3) and the “Survey” package was used for parameter estimation.

## Results

### Demographic Characteristics of Study Subjects

Parkinson’s disease and controls were similar in age, gender, history of smoking, and statin usage (Table 1). Total- and LDL-cholesterol levels were significantly lower in the PD group (*p* = 0.03 and 0.04, respectively), whereas HDL and triglycerides were similar in the two groups. The majority of both control (80%) and PD (86%) subjects had the ε3ε3 phenotype, with the remaining control and PD subjects having the ε3ε2 phenotype. One PD subject had the ε2ε2 phenotype, and no subjects had an ε4 allele. PD and controls had similar proportions of the two LDLR SNPs investigated in our study (*p* > 0.10). In addition, there was no difference in the proportion of the three LRP1 SNPs between PDs and controls (*p* > 0.19), or the LRPAP1 SNP (*p* = 0.24).

### The Effects of Age, Gender, Smoking, Statin Usage, and Genes on Blood Cholesterol Levels

**Figure 1:**
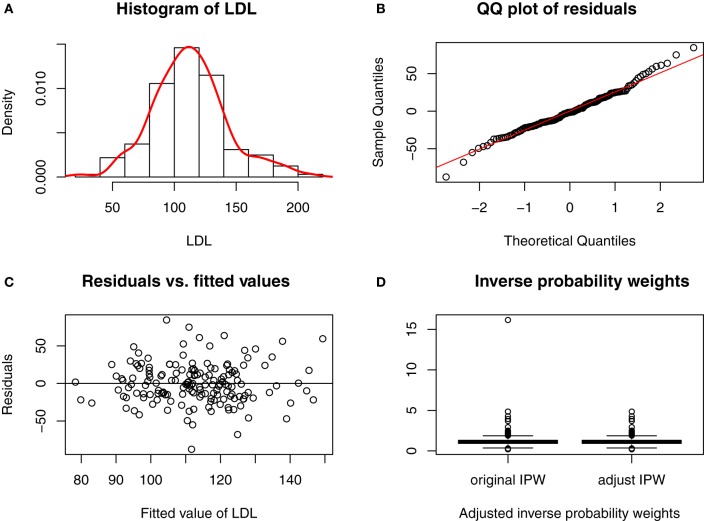
Model diagnostic for fitted model in the causal link of LDL-cholesterol to PD risk. **(A)** Histogram of LDL. **(B)** QQ plot of residuals. **(C)** Residuals vs. fitted values. **(D)** Inverse probability weights.

Age and history of smoking did not influence significantly cholesterol levels in our cohort, whereas PD subjects had lower total- and LDL-cholesterol (Table 1). The histogram in Figure 1A and the QQ plot in Figure 1B show that LDL is approximately normally distributed (total-cholesterol also has an approximately normal distribution, not shown). We conducted multiple regression using our initial model (Table 2, Model I) that included APOE and six SNPs in the LDLR, LRPI, and LRPAP1 genes, and adjusted for several potential confounding variables, including smoking history, statin use, age, and gender. We also performed model selection based on the Akaike information criterion (AIC, stepAIC function in R) for total- and LDL-cholesterol, referred to as Model II in Table 2. Figure 1C shows that the residuals from fitted values for LDL in Model II from Eq. 3 have approximately constant variance and are normally distributed (Shapiro–Wilk test has *p* = 0.1).

**Table 2:** Confounder effects on cholesterol levels.

	Total-cholesterol				LDL–cholesterol			
	Model I^a^		Model II^b^		Model I^c^		Model II^d^	
Cofounder	Estimation β (SD)	*p*	Estimation β (SD)	*p*	Estimation β (SD)	*p*	Estimation β (SD)	*p*
Age (years)	−0.20 (0.24)	0.40	−0.16	0.48	−0.18 (0.2)	0.39	−0.16 (0.2)	0.42
Gender (M)	−16.44 (5.38)	0.003*	−17.27	0.001*	−4.83 (4.62)	0.30	−5.71 (4.57)	0.21

**Statin and smoking effects on cholesterol levels**								

Statin usage (Yes)	−20.96 (5.31)	<0.001*	−20.49	<0.001*	−19.90 (4.75)	<0.001*	−19.31 (4.71)	<0.001*
Smoke (Yes)	−9.64 (5.87)	0.10	−8.97	0.12	−6.30 (5.04)	0.21	−5.55 (4.94)	0.26

**Genes related to cholesterol management**								

APOE polymorphism	12.07 (7.14)	0.09	12.24	0.08	10.69 (6.13)	0.08	10.71 (6.06)	0.08

**LDLR single-nucleotide polymorphisms (SNPs)**								

rs6511720 (GG TG TT)	−21.45 (22.54)	0.34	NA	NA	−25.22 (19.36)	0.19	NA	NA
rs2228671 (CC TC TT)	10.35 (24.0)	0.67	NA	NA	16.88 (20.62)	0.41	NA	NA

**LRP1 SNPs**								

rs11172113 (CC CT TT)	−22.83 (11.17)	0.04*	−24.6	0.027*	−19.83 (9.59)	0.04*	−21.8 (9.46)	0.023*
rs1466535 (CC TC TT)	−17.91 (12.14)	0.14	−18.76	0.12	−17.22 (10.43)	0.10	−18.26 (10.33)	0.08
rs1800127 (CC TC TT)	8.48 (19.20)	0.66	NA	NA	6.27 (16.49)	0.70	NA	NA

**LRPAP1 SNPs**								

rs11549516 (CC TC TT)	36.62 (25.40)	0.15	40.69	0.1	37.39 (21.82)	0.09	42.19 (21.39)	0.05

*^a^Total-cholesterol: Model I: AIC = 1,586.1*.

*^b^Total-cholesterol: Model II after model selection: AIC = 1,582.1*.

*^c^LDL-cholesterol: Model I: AIC = 1,537.2*.

*^d^LDL-cholesterol: Model II after model selection: AIC = 1,533*.

*Significant based on Wald tests.

As expected, statin usage was associated significantly with lower total- and LDL-cholesterol levels (Table 2). Among the genes we investigated, before model selection only LRP1 demonstrated a statistically significant influence on total- and LDL-cholesterol levels (*p* = 0.04 for both in Model I, and *p* = 0.0027 and *p* = 0.024 separately in Model II). LDLR SNPs did not significantly impact cholesterol levels and were not selected in Model II after step-wise model selection. There was a trend for APOE polymorphisms to influence both total- and LDL-cholesterol (*p* = 0.08 and *p* = 0.08, respectively), and also a trend of LRPAP1 influencing LDL-cholesterol (*p* = 0.05).

### The Causal Effect Analyses of Total- and LDL-Cholesterol Levels on PD Using the Generalized Propensity Score Approach

To assess the causal relationship between cholesterol and PD, a marginal structure model with generalized propensity score approach was adopted. This model can be employed for more general settings than structural equation modeling (SEM) (33), and more importantly, it involves fewer assumptions than SEM because it targets a particular set of effects of interest, and only makes assumptions needed for those effects (28, 34–37).

Figure 1D shows the boxplot of the IPW for LDL-cholesterol, and there is one extreme weight with a value greater than 10. This extreme value can affect the variance of causal estimates (38), and thus we shrank it to the 95th quantile as an adjustment for the IPW. Total-cholesterol had two extreme weights greater than 10, and we shrank these two values to the 95th quantile as well.

Both total- and LDL-cholesterol levels were significantly linked to PD occurrence through the generalized propensity score method. The estimates of effects are −0.032 (SE: 0.009) with *p* < 0.001 for total-cholesterol, and −0.016 (SE: 0.008) with *p* = 0.04 for LDL-cholesterol, respectively. The odds ratios are 0.97 [95% CI: 0.95, 0.99] and 0.98 [95% CI: 0.97, 0.99] for total- and LDL-cholesterol, respectively.

## Discussion

Despite a growing literature implying that serum/plasma total- and LDL-cholesterol may be linked to PD (1–12), the cause of the association is not known. In the current study, we applied the generalized propensity score approach to investigate a potential causal relationship between circulating cholesterol levels and PD. SEM is an alternative tool for causal inference, but generalized propensity score with inverse probability of weights is a new class of causal model that can handle more challenges (e.g., time-dependent confounding) with fewer assumptions than SEM. Following the convention in the literature of causal inference (28), the method accounts for confounding effects through a linear combination of genetic, biological, behavioral, and environmental factors. Due to the small set of genes for consideration, there is no need to conduct a boosting algorithm for propensity score estimation in our case, and we assumed a normal distribution for the multiple linear regressions shown in Figure 1A.

We took into consideration age; gender; APOE polymorphisms; LDLR, LRP1, and LRPAP1 SNPs; smoking history; and statin usage in our initial model (Model I) because they are suspected to influence both circulating cholesterol levels and the occurrence of PD. After step-wise model selection, SNPs from LDLR were removed in Model II. The results of these analyses suggest that circulating total- and LDL-cholesterol levels may be linked to the factors influencing PD risk.

### The Effect of Smoking, Statins, and Cholesterol-Related Genes on Cholesterol Levels

Our analyses, as expected, found that statins lower cholesterol and lower cholesterol (after controlling for statin use) could be a risk factor for PD, in agreement with the large majority of the literature (1–12). Diabetes may affect lipid levels, and statins have been suggested to be of use in diabetes (39), but because this is not clearly established, diabetes was not included in our analyses. The literature related to the role of smoking on cholesterol levels has been mixed, with some studies reporting an association, but others not (40, 41). In our current cohort, history of smoking did not influence cholesterol profiles significantly, although this may have been affected by our categorization of smokers as those who had smoked consistently for six months at any time in their life. The lack of an association of age and cholesterol levels probably resulted from the very narrow age range of the subjects in the study (i.e., PD is a disorder of later life and the controls were age-matched).

The most common allele in APOE, ε3, is present in ~77% of the population, whereas the least common ε2 is represented in only 8%. Individuals with an ε2 allele have been known to have a propensity for lower plasma LDL-cholesterol levels, whereas ε4 is linked to higher LDL-cholesterol levels (42). Although ε2 is linked to a number of beneficial outcomes in terms of cardiovascular disease and lower risk of AD, some studies (19, 20), but not all in others (43), have associated it with higher risk of PD. Conversely, the ε4 allele is associated with poorer cardiovascular disease outcomes and a significantly increased risk of AD (44, 45), but may be protective and/or associated with lower PD risk (21). Although there was a trend for APOE to influence cholesterol levels in the current study, the analysis did not reach statistical significance, probably due to the small sample size and the lack of ε4 alleles in this cohort. Since the ε4 allele occurs in ~14% of the general population (46), it is somewhat remarkable that no subject in the study had an ε4 allele. This may be due to the lower occurrence of ε4 in PD (21), and the fact that the Controls, by being “healthy” (i.e., no cardiovascular disease or AD), would tend not to be ε4 carriers.

### A Potential Causal Cholesterol–PD Link

The current study suggests for the first time that circulating total- and LDL-cholesterol may be linked to the factors influencing PD risk, but it is not informative about the specific mechanism or factor(s) that mediate such a link. Statins have been suggested to be neuroprotective for PD (47–49). The current study, however, suggested that statins, by lowering cholesterol, actually may be linked to PD risk. Consistent with this latter hypothesis, a prospective study in the Atherosclerosis Risk in Community cohort found that statin usage was associated with increased future risk of PD (9). Most recently, an analysis of the large MarketScan national claims database found that statins facilitated PD diagnosis (50). Together, these data suggest caution to proposing statins as being neuroprotective for PD.

The brain is the most cholesterol-rich organ in the body (accounting for ~25% of the total amount). In mature brain, cholesterol is synthesized primarily by astrocytes and then transported to neurons *via* endocytosis and interaction with the LDL receptor and apolipoprotein E (51). There is very limited ability for circulating cholesterol to traverse the blood–brain barrier (BBB) (52). Thus, according to current thinking (53), brain cholesterol is made mainly de novo, and central nervous system (CNS) and plasma compartments generally do not communicate (54). There is, however, evidence for the uptake of LDL particles and other apolipoproteins across the BBB, possibly *via* the LDLR and/or LDLR-related proteins, and oxysterols also may mediate peripheral-central cholesterol communication (55).

Genetic defects in cholesterol metabolism (i.e., lipoprotein receptors, cholesterol biosynthesis, lipid transport and assembly, or signaling molecules) may lead to structural and functional CNS diseases (53), and many cholesterol metabolites are centrally active (56). Cholesterol synthesis in CNS can be regulated by the APOE-mediated uptake of lipoproteins via the LDLR family of proteins. After synthesized cholesterol is delivered to neurons, it is bound by cell surface lipoprotein receptors such as the LDLR receptor and the LDLR-related protein (LRP1) that recognize APOE prior to endocytosis.

It is also possible that LRP1 mediates a compensatory response to neural stress and elicits a parallel change in LDL particles and other apolipoproteins across the BBB *via* the LRP receptor. MPTP lesions increase both APOE and its receptors (LDLR and LRP) in brain (23). APOE and LRPAP1 SNPs are associated significantly with increased PD risk, and high levels of LDL-cholesterol appear to have a protective role against PD (22). Consistent with this notion, our results showed that the LDLR-related gene LRP1 had a significant influence on circulating total- and LDL-cholesterol levels. These lines of evidence may seem paradoxical, but can be reconciled. The synthesis of cholesterol, its oxidation products, and APOE are increased in response to neural cell injury, thus an increase in brain cholesterol and CSF levels of LRP1 could be compensatory responses to neural stress.

### Limitations and Future Directions

There are 152 cholesterol-related genes and more than 3,000 SNPs covered in the NeuroX array based on plasma lipid studies in coronary artery disease (26). In the current study, we investigated only four genetic polymorphisms potentially linked to both cholesterol profiles and neurodegenerative processes (18). As such, further studies in larger samples are needed to investigate other potentially important cholesterol-related genes and SNPs that also may be involved in neurodegenerative processes. Although our study suggested a potential causal link between circulating cholesterol and PD, there are no clear mechanistic explanations for this finding. Future studies are needed to provide mechanistic understanding and shall include a revisit of the presumed segregation of peripheral and central cholesterol, especially in pathological states and/or aging.

## Ethics Statement

This study was carried out in accordance with the recommendations of XH’s NIH PD Biomarkers Program at the Penn State site, with written informed consent from all subjects. All subjects gave written informed consent in accordance with the Declaration of Helsinki. The protocol was approved by the Penn State Hershey Institutional Review Board.

## Author Contributions

LZ: organization, execution of the project; statistical design and execution; writing of the first draft; and review and critique of the manuscript. XW: statistical design and execution, and review and critique of the manuscript; MW: review of statistical design and review and critique of the manuscript. NS: organization of the project and review and critique of the manuscript. GD: organization of the project; review and critique of the statistical design; and critique of the manuscript. ML: organization of the project and extensive review and critique of the manuscript. TY: execution of the project; statistical design and execution; and review and critique of the manuscript. RM: review of statistical design and extensive review and critique of the manuscript. RL: conception, statistical design and execution, and review and critique of the manuscript; XH: conception, organization, execution, obtaining funding of the project; review of the statistical design; writing of the first draft; and review and critique of the manuscript.

## Conflict of Interest Statement

The authors declare that the research was conducted in the absence of any commercial or financial relationships that could be construed as a potential conflict of interest. The reviewer PV-C and handling editor declared their shared affiliation.

## Abbreviations

APOE, apolipoprotein E gene; CNS, central nervous system; LDLR, low-density lipoprotein receptor gene; LDLR1, low-density lipoprotein receptor related protein 1 gene; LRPAP1, low-density lipoprotein-related protein-associated protein 1 gene; BBB, blood–brain barrier.
